# Carbon Nanotube Reinforced Poly(ε-caprolactone)/Epoxy Blends for Superior Mechanical and Self-Sensing Performance in Multiscale Glass Fiber Composites

**DOI:** 10.3390/polym13183159

**Published:** 2021-09-18

**Authors:** Xoan F. Sánchez-Romate, Andrés Alvarado, Alberto Jiménez-Suárez, Silvia G. Prolongo

**Affiliations:** Materials Science and Engineering Area, Escuela Superior de Ciencias Experimentales y Tecnología, Universidad Rey Juan Carlos, Calle Tulipán s/n, Móstoles, 28933 Madrid, Spain; andresalvafranc@gmail.com (A.A.); silvia.gonzalez@urjc.es (S.G.P.)

**Keywords:** smart materials, damage detection, carbon nanotubes, multiscale composites, interlaminar properties

## Abstract

In this paper, a novel carbon nanotube (CNT) polycaprolactone (PCL), epoxy, and glass fiber (GF) composite is reported. Here, the nanoreinforced composites show a flexural strength increase of around 30%, whereas the interlaminar shear strength increases by 10–15% in comparison to unenhanced samples. This occurs because the addition of the CNTs induces a better PCL/epoxy/GF interaction. Furthermore, the nanoparticles also give novel functionalities to the multiscale composite, such as strain and damage monitoring. Here, the electrical response of the tensile- and compressive-subjected faces was simultaneously measured during flexural tests as well as the transverse conductivity in interlaminar tests, showing an exceptional capability for damage detection. Moreover, it was observed that the electrical sensitivity increases with PCL content due to a higher efficiency of the dispersion process that promotes the creation of a more uniform electrical network.

## 1. Introduction

Fiber-reinforced polymers are gaining a great deal of attention over common structural metals. More specifically, glass fiber reinforced polymers (GFRPs) offer an attractive potential for many applications, such as marine infrastructures [1] or wind turbine blades [2,3], due to their efficient mechanical performance and corrosion resistance.

However, most structural composites, specifically GFRPs, are based on rigid and brittle resins such as epoxy or polyester. Therefore, it is necessary to investigate possible toughening mechanisms in these kinds of systems to make them more suitable for both structural and miscellaneous applications. In this regard, the use of thermoplastic blends, such as (poly)caprolactone (PCL), has effectively promoted these toughening mechanisms [4,5]. This effect occurs because PCL is miscible in the epoxy matrix, but a phase separation takes place during curing, leading to a restriction of the toughening phase.

The toughening effect of the PCL has been widely explored in reinforced polymers. More specifically, van der Heijden et al. [6] observed an improvement of almost 100% in the fracture toughness when using PCL nanofibers. In addition, the toughening mechanisms of PCL were also observed by Daelemans et al. [7] in an enhancement in the damage resistance under low velocity impacts.

Furthermore, PCL blends show other interesting functionalities. For example, they can act as healing agents; the healing mechanism is based on the flow of the thermoplastic phase to fill a crack when subjected to higher temperatures than its melting point. In this context, many studies have observed high healing efficiencies when subjected to fracture energy and impact tests [8,9], leading to an increase in the lifetime of the composite structure.

This study aims to analyze an effect that remains unexplored in scientific literature: the addition of carbon nanoparticles, specifically carbon nanotubes (CNTs), in multiscale epoxy/PCL/GF composites. In this regard, the addition of CNTs promotes the creation of a percolating network inside of the matrix. This leads to a drastic increase in its electrical conductivity of several orders of magnitude [10,11,12]. Therefore, the addition of CNTs gives the composite new and interesting multifunctionalities. More specifically, the composite shows excellent piezoresistive behavior that, in conjunction with the tunneling transport that occurs between adjacent nanoparticles, makes possible the strain and damage monitoring by means of electrical conductivity measurements [13,14,15,16,17]. This capability has been extensively explored in multiscale GF composites [18], showing a particularly high sensitivity to interlaminar [19], impact failure [20], or moisture damage propagation [21], as well as good flow and cure monitoring capabilities [22,23], making the multiscale composites suitable for structural health monitoring (SHM) applications. Furthermore, the damage sensing capabilities have been explored in relatively complex geometries under electrical impedance tomography (EIT) [24] and in other types of materials, such as adhesive joints [25].

Moreover, the addition of the CNTs promotes the enhancement in interlaminar and fracture properties in multiscale materials. It has been observed that their presence improves the mode-II fracture toughness of graphite-UD and GFRP composites [26,27]. In addition, they have shown a high strengthening effect in bonded joints for wind blades [28,29].

The influence of CNT addition on the physical properties and mechanical performance of PCL/epoxy blends is gaining interest. Previous studies have analyzed their effect on the glass transition temperature, miscibility, and electromechanical performance at the nanocomposite level [30,31]; however, their effect at the multiscale composite level remains to be investigated.

Therefore, this work is focused on taking advantage of both the toughening and interlaminar enhanced properties of PCL blends in multiscale composites, alongside the SHM capabilities of the CNTs. In this regard, flexural and interlaminar properties of the CNT-reinforced PCL/epoxy/GF composites are analyzed, with special attention given to the microstructure of PCL blends. In addition, the electrical conductivity of multiscale composites is investigated in both transverse and longitudinal direction to explore the influence of PCL content and directionality. Finally, SHM capabilities are widely explored by the simultaneous measuring of the electrical conductivity of the composites, to correlate the mechanical and electrical responses for damage detection. The main purpose of this study is to develop enhanced mechanical multiscale composites with added functionalities.

## 2. Materials and Methods

### 2.1. Materials

The matrix used in this study was an epoxy resin Araldite LY556 with a hardener, XB 3473, both supplied by Hustman (The Woodlands, TX, USA), as well as (poly)caprolactone with a molecular weight of 80,000 g/mol, purchased from Sigma Aldrich (St. Louis, MS, USA), which was grained in order to facilitate its dissolution in the epoxy resin.

The reinforcement was an E-glass fiber non-crimp fabric supplied by Resinas Castro (Pontevedra, Spain). The multiscale composite was manufactured following a stacking sequence of [0/90]_4s_.

*NC7000* multi-wall carbon nanotubes (MWCNTs) with an average diameter of 9.5 nm and a length of up to 1.5 μm were supplied by Nanocyl (Sambreville, Belgium). 

### 2.2. Manufacturing Process

Multiscale glass fiber composites were fabricated using a manual lay-up process and cured in a hot press. Prior to the composite manufacturing, MWCTNs were dispersed in the PCL/epoxy blends.

The dispersion procedure consisted of several steps: (1) PCL was dissolved in the epoxy matrix at 80 °C following a 10 min magnetic stirring process. (2) MWCNTs were mixed with the PCL/epoxy blends then dispersed through a three roll milling process at 80 °C; this cycle was repeated seven times, with a progressive reduction in the gap between rolls. These parameters were optimized in a previous study [32] and are shown in Table 1. (3) After the dispersion procedure, the mixture was degassed in vacuum conditions at 80 °C, and the hardener was added in at a ratio of 100 to 23.

After the mixture was prepared, it was manually applied, layer by layer, over the GF by using a compaction roll in order to remove any entrapped air. Finally, after the manual lay-up process, the plates were cured in a hot-press at 140 °C for 8 h at a pressure of 0.6 MPa. A summary of the different manufactured conditions is shown in Table 2.

### 2.3. Electromechanical Tests

The obtained CNT/PCL/epoxy/GF composites were subjected to three-point bending and interlaminar shear strength tests. The flexural tests followed the ASTM D790 at a test rate of 1.2 mm/min, on 100 mm × 13 mm × 3.5 mm samples. ILSS tests were conducted according to ASTM D234 at a crosshead speed of 1 mm/min, on 24 mm × 8 mm × 3.5 mm samples. Six specimens were tested for each condition.

**Figure 1:**
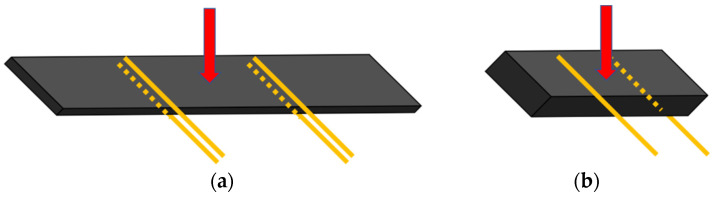
Schematics of electrode disposition in flexural (**a**) and ILSS (**b**) tests, where the red arrow denotes the direction of the applied load.

Simultaneous to mechanical testing, the electrical response was recorded with an Agilent 34410A module (Agilent Technologies, Santa Clara, CA, USA) to prove the SHM capabilities of the proposed multiscale composites. To achieve this, the electrical resistance was measured between several electrodes made of copper wire and attached to the composite surface with silver ink. The schematics of the electrode’s disposition are shown in Figure 1. The electrical sensitivity (also called gauge factor), *S*, is calculated by the change in the normalized resistance $\frac{\Delta R}{R_{0}}$ divided by the applied strain, *ε*:

(1) $$S=\frac{\frac{\Delta R}{R_{0}}}{\varepsilon }$$

### 2.4. Characterization of Multiscale Composites

The electrical conductivity in the transverse and longitudinal directions of GF multiscale composites was measured using the I-V curves according to ASTM D257-14 in 100 mm × 13 mm × 3.5 mm and 40 mm × 40 mm × 3.5 mm samples, respectively. The electrical measurements were carried out using a Keithley 2410 multimeter (Keithley Instruments, Solon, OH, USA). Six specimens of each condition were tested for this purpose.

Moreover, a microstructural analysis of fracture surfaces was carried out in order to better understand the morphology of PCL/epoxy blends and to analyze the prevalent failure mechanisms. A detailed analysis of crack propagation was performed through scanning electron microscopy (SEM) by using a Hitachi S-3400 N apparatus (Hitachi, Ltd., Tokyo, Japan). Prior to observation, each sample was coated with a layer of gold. The analysis of prevalent failure mechanisms for the correlation with the electrical measurements was conducted through optical microscopy, using a *Leica* apparatus (Leica Camera, Wetzlar, Germany), equipped with a *Nikon Coolpix 990* camera (Nikon Inc., Tokyo, Japan).

## 3. Results

In this section, an analysis of mechanical properties is carried out. Subsequently, the electrical and electromechanical properties are deeply discussed by identifying the main conducting mechanisms and the SHM applicability of the proposed materials.

### 3.1. Mechanical Analysis

**Figure 2:**
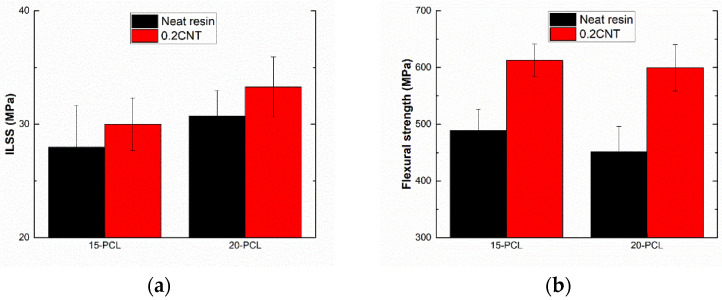
(**a**) ILSS and (**b**) flexural strength of GFRP samples at the different tested conditions.

Figure 2 summarizes the ILSS and flexural strength values of the GFRP and PCL samples under the different tested conditions. First, it can be observed that ILSS increases with both PCL and CNT content. This effect correlates with the synergistic effects of both PCL and CNT on the interlaminar strength of composite laminates.

**Figure 3:**
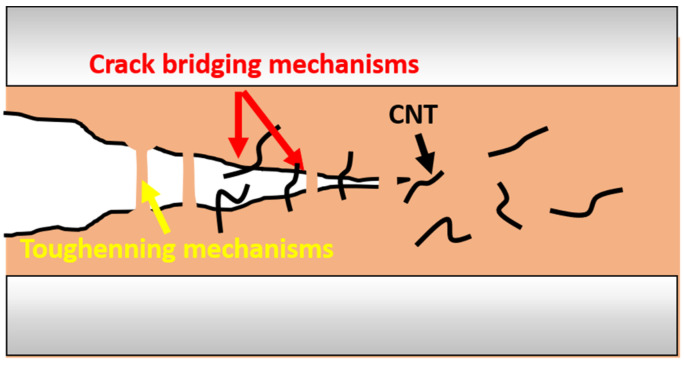
Schematics of crack-bridging mechanisms in CNT/PCL/epoxy multiscale composites.

On one hand, it has been observed that CNT addition also induces an enhancement in interlaminar properties due to the crack-bridging effect (Figure 3) promoted by the nanoparticles [33,34,35], leading to a toughness enhancement. On the other hand, it has been proven that the addition of a thermoplastic phase, such as PCL, promotes an interlaminar toughening [6,36]. In this regard, PCL is miscible with several amine-cured epoxy resins [37], while a two-phase morphology was observed in other similar resins [38,39], which are currently being examined due to their self-healing ability. In addition, PCL is proposed as a toughening modifier of epoxy resins with phase inversion.

Phase-separated PCL/epoxy blends are obtained through phase separation and induced by chemical reaction. In this case, PCL is soluble into the mixture of monomers, but it becomes immiscible as the epoxy molecular weight increases during the curing reaction process. Phase separation involves several processes while it cures: onset of phase separation, gelation, growth and fixation of the phase separated size, end of phase separation, and vitrification. Phase separation can be inhibited when the blend is at high viscosity, gelation, or vitrification.

In the particular case of PCL/epoxy blends, there are other phenomena implied, such as the formation of hydrogen bonding between the ester groups of the thermoplastic with the hydroxyls of the epoxy network [37,40]. These hydrogen bonds increase the PCL miscibility, thus causing phase separation to occur at high conversion rates [41]. When the theoretical phase separation conversion is higher than the gel point, a homogenous blend is obtained. This transesterification reaction induces the excision of PCL chains; these segments are grafted onto the epoxy network, resulting in a homogenous blend.

**Figure 4:**
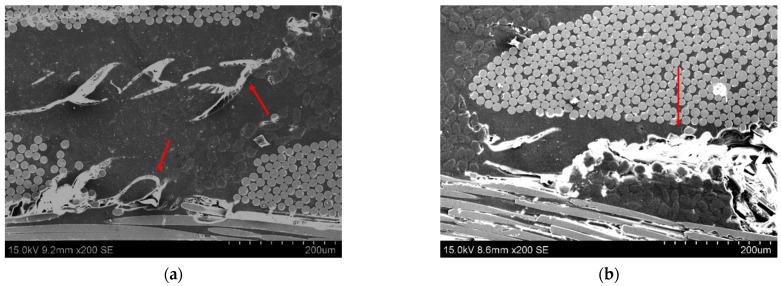
SEM images of transversal sections of (**a**) 15PCL-02CNT and (**b**) 15PCL-Neat.

Figure 4 shows several SEM images of transversal sections of ILSS samples. Here, there is no phase separation between the epoxy matrix and the PCL. Therefore, a homogeneous blend was obtained, leading to a significant enhancement in the toughness of the system, as observed in Figure 3. Moreover, the CNTs promote difficult crack propagation in combination with the PCL, resulting in much more tortuous crack paths due to the presence of the nanofillers [42] (red arrows in Figure 4a), whereas the samples without nanoreinforcement present a more prevalent interlaminar failure (red arrow in Figure 4b), which would explain the lower ILSS. Therefore, the microstructural analysis proves the synergetic effect of both CNTs and PCL in the interlaminar properties.

The flexural strength significantly increased with the addition of CNTs, whereas a slight decrease was observed with increasing PCL content (Figure 2b). This detriment was more prevalent in the neat samples (without CNTs), while the multiscale samples (with a 0.2 wt.%) were hardly affected by increasing the PCL content. This behavior can be explained by the opposite effect induced by both the CNTs and PCL. On one hand, the addition of carbon nanotubes may enhance the interfacial adhesion between the matrix and the fiber, leading to a more effective load transfer, thus increasing mechanical properties [43]. On the other hand, PCL addition may negatively affect the flexural properties of the composite due to its lower mechanical performance. In this regard, a higher miscibility and better distribution of the thermoplastic phase could inhibit the detriment in the mechanical performance due to a better combination of the blends and the glass fibers [44]. As previously mentioned, the addition of carbon nanoparticles promotes a higher miscibility of the PCL in the epoxy matrix, which explains the decline in mechanical properties when increasing the PCL content. 

### 3.2. Electromechanical Analysis

First, the electrical conductivity in the longitudinal and transverse directions was analyzed. Then, the electromechanical properties under flexure and ILSS tests were also explored in order to prove the SHM capabilities of the proposed materials.

#### 3.2.1. Electrical Conductivity Measurements

**Figure 5:**
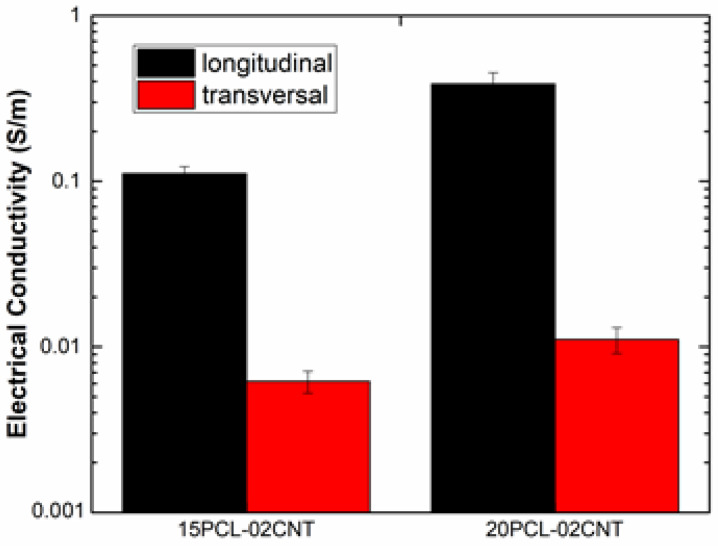
Electrical conductivity values of the PCL/CNT GFRP samples in the longitudinal and transverse direction.

Figure 5 shows the electrical conductivity of the GFRP/PCL/CNT samples in the longitudinal and transverse directions. The samples without carbon nanoparticles were not electrically conductive.

**Figure 6:**
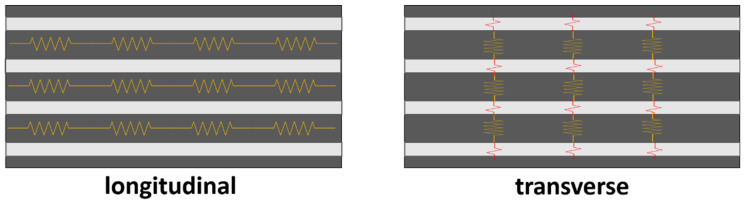
Schematics of the main electrical pathways in the longitudinal and transverse directions of the GFRP laminate.

First, it can be observed that the electrical conductivity is significantly higher compared to previous studies with multiscale GF composites reinforced with graphene nanoplatelets (GNPs) [45]. This is explained by the much lower percolation threshold of CNTs [46] that promotes the creation of a very effective electrical network inside the material. In addition, the electrical conductivity in the longitudinal direction is more than one order of magnitude higher than in the transverse direction. This anisotropic behavior was observed in other studies [45], and is explained by the disposition of the glass fiber in the laminate, and how it affects the electrical network. In this regard, Figure 6 shows a schematic of the electrical contacts in the longitudinal and transverse directions. Here, the GF has a more prevalent role in the transverse direction and, due to its insulating nature, induces a detriment of the electrical conductivity.

It can be observed that the electrical conductivity increased with the PCL addition. This effect was also observed in a previous study in PCL and CNT nanocomposites [31]. It is explained by the higher efficiency of the dispersion procedure when increasing the amount of PCL. More specifically, the higher viscosity of the PCL promotes a higher prevalence of the shear forces during the three roll-milling processes, leading to a more significant disaggregation of agglomerates and thus, a more efficient CNT network, which is reflected in the increase in the electrical conductivity.

#### 3.2.2. Electromechanical Tests

**Figure 7:**
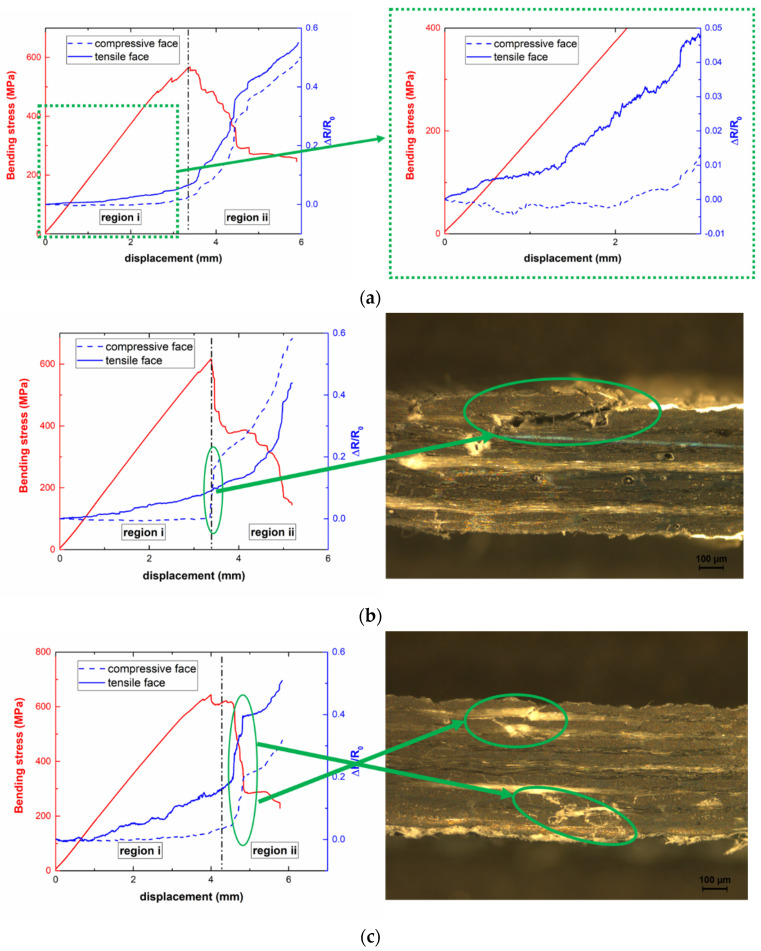
Electromechanical response of (**a**) 15PCL-02CNT and 20PCL-02CNT samples with (**b**) a prevalent compressive failure and (**c**) mixed tensile-compressive failure, where the micrographs on the left denote the transversal sections.

Figure 7 shows some representative examples of the electromechanical response under flexural load. Here, the blue lines represent the tensile (solid lines) and compressive (dashed lines) subjected faces of each sample.

The usefulness of the bending tests lies in the fact that they provide a more realistic scenario than uniaxial tensile tests, as one possible application, such as wind blades, is subjected to bending moments. In addition, it would allow the evaluation of the SHM capability of the proposed system in a more complex load state.

Two main regions can be distinguished during the bending test. The first region (region i) corresponds to the initial coupon deformation due to the bending stress before failure; a second region (region ii) corresponds to the bending failure of the sample. Regarding region i, the behavior is slightly different depending on the compressive- or tensile-subjected face. In the compressive subjected face, an initial, slight decrease in the electrical resistance is observed at low strain levels, followed by an increase in the electrical resistance at higher strain values. This behavior was previously reported [47] and is correlated to an initial reduction in the tunneling distance between adjacent nanoparticles, followed by the nucleation of microcracks that explain the subsequent increase in the electrical resistance. Furthermore, the electrical resistance of the tensile subjected face monotonously increases following a typical linear-exponential behavior due to the tunneling mechanisms involved [48].

Table 3 shows the values of the sensitivity, *S*, calculated from Equation (1) at ε=0.015 for the tensile- and compressive-subjected faces at the different PCL/CNT conditions. It can be observed that an increase in PCL content promotes an increase in the electrical sensitivity. This effect can be attributed to a better CNT dispersion due to the aforementioned higher efficiency of the three roll milling process, which promotes a higher prevalence of tunneling transport mechanisms over contact ones [49]. In addition, the sensitivity of the compressive face at this strain level is much lower, as expected, than the sensitivity of the tensile face, in some cases leading to negative values.

The electrical response in the second region ii may be different depending on the failure type. Here, Figure 7b,c shows two different electromechanical responses. On one hand, Figure 7b shows a sharp increase in the electrical resistance in the compressive-subjected face, whereas the electrical response in the tensile-subjected face is not significantly affected. This may be indicative of a preferential compressive failure, as there is a sudden breakage of electrical pathways in the associated channel. This hypothesis is confirmed by the microscopic analysis of the transversal sections after failure where a prevalent matrix cracking and fiber breakage was observed in the compressive-subjected face, whereas no prevalent failure mechanisms were observed in the tensile face (micrograph of Figure 7b. On the other hand, Figure 7c shows a similar electromechanical response in both compressive and tensile channels, indicating a more homogeneous failure that affects both sides of the specimen, which is confirmed by the analysis of transversal sections (micrographs of Figure 7c). Therefore, the electrical monitoring gives detailed information about the location of the damage, and subsequently, the failure type.

**Figure 8:**
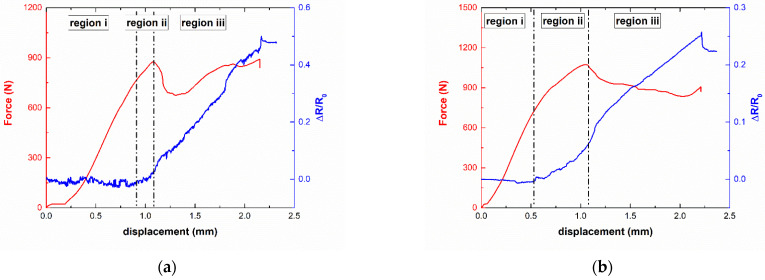
Electromechanical curves of ILSS (**a**) 15PCL-02CNT and (**b**) 20PCL-02CNT samples.

Figure 8 shows some representative examples of the electromechanical response under ILSS tests. Here, it can be observed in three different regions: (i) the initial stage, which is reflected in a linear increase of the applied load and an almost constant electrical response due to the absence of crack propagation mechanisms; (ii) a second stage that corresponds to the initial nucleation of the crack, which is reflected in a loss of stiffness and a soft increase in the electrical resistance due to the breakage of electrical pathways; and (iii) a final stage that corresponds to the interlaminar crack propagation, reflected in a sudden drop in the applied load, and a subsequent sharp increase in the electrical resistance due to a rapid breakage of electrical pathways. Therefore, a good agreement between the electrical and the mechanical response is noticed.

Several differences can be observed when comparing the samples with 15 and 20 wt.% PCL. More specifically, the increase in PCL content is reflected in a smaller drop of the applied load (region iii in Figure 8b) and a subsequent smaller increase in the electrical resistance (a variation in the normalized resistance of 25% at the end of the test in comparison to 50% in the 15 wt.% PCL samples) due to the interlaminar toughening mechanisms of the PCL, as well as its increasing miscibility with CNT. Furthermore, it can be observed that region ii, which corresponds to the initial crack nucleation in the material, is more prevalent in the case of 20 wt.% PCL samples. The toughening effect of the PCL and the higher miscibility induced by the CNT addition promotes a softer nucleation of the cracks inside the material. In this regard, the electrical resistance can confirm the crack propagation mechanisms taking place in these types of materials.

## 4. Conclusions

The mechanical and electrical properties of CNT-reinforced PCL/epoxy/GF multiscale composites were widely explored.

It was observed that the addition of CNTs promotes a significant enhancement of around 30% in the flexural strength. Moreover, the ILSS increased by 10–15%. This is explained by the toughening and crack-bridging mechanisms of CNTs in combination with the effect of miscible PCL.

In addition, the values of the electrical conductivity are superior to other nanoreinforced multiscale composites. More specifically, an increasing amount of PCL promotes an enhancement in conductivity due to its effect on the dispersion of CNTs. In this regard, the higher viscosity of PCL leads to a higher efficiency of the dispersion procedure, promoting the creation of a more uniform and efficient electrical network.

The SHM capabilities of CNT-reinforced PCL/epoxy/GF composites were proved. More specifically, it was observed that the electromechanical response during flexural tests depends on the location of the electrodes, as well as the location of the prevalent failure. Here, the electrical response of compressive-subjected face is less sensitive to applied strain than the tensile face at the first stage of the flexural test due to the approaching effect of adjacent nanotubes. Furthermore, the prevalent failure mechanisms induce a sharp increase in electrical resistance due to the sudden breakage of the electrical pathways. In this regard, by analyzing the electrical responses of both compressive and tensile faces, it is possible to locate the region of prevalent failure.

A notable correlation between electrical and mechanical response was observed in ILSS tests by identifying the moment at which the first crack nucleation takes place. Therefore, enhanced mechanical and self-sensing GFRPs were developed by the addition of novel CNT-reinforced PCL/epoxy blends.

## Figures and Tables

**Table 1:** Three roll-milling parameters.

Cycle	Distance between Rolls (GAP1/GAP2 in µm)
Cycle 1	120/40
Cycle 2	60/20
Cycle 3	45/15
Cycles 4 to 7	15/5

**Table 2:** Nomenclature used for the different manufactured multiscale composites.

PCL Content (wt.%)	MWCNT Content (wt.%)	Designation
15	0	15PCL-Neat
	0.2	15PCL-0.2CNT
20	0	20PCL-Neat
	0.2	20PCL-0.2CNT

**Table 3:** Sensitivity values at the compressive and tensile face for CNT-reinforced PCL/epoxy/GF composites during the bending test.

Condition	Sensitivity, *S*, at *ε* = 0.015	
	Tensile Face	Compressive Face
15PCL-02CNT	1.1 ± 0.5	0.2 ± 0.2
20PCL-02CNT	3.0 ± 0.8	−0.1 ± 0.2

## Data Availability

The data presented in this study are available on request from the corresponding author.
